# Efficiency of a psychiatric support for PTSD on a cohort of relatives of patients hospitalized in intensive care unit during the French lockdown – the OLAF (Opération Liaison et Aide aux Familles in French) dispositive

**DOI:** 10.1192/j.eurpsy.2023.981

**Published:** 2023-07-19

**Authors:** V. Raymond, J. Salles, C. Aïtout, G. Ducos, S. Silva, C. Arbus, P. Birmes

**Affiliations:** Psychiatry; 2 Anesthesiology, Toulouse University Hospital, Toulouse, France

## Abstract

**Introduction:**

Having a family member admitted in Intensive Care Unit (ICU) can cause a severe psychological stress, and various psychological disorders gathered into the notion of Post intensive care syndrome-family (PICS-F). In this way, Family-centered care in ICU represents the aim of international accepted recommendations focusing on a partnership approach to health care decision-making between the family and health care provider to prevent PICS-F. During the first SARS-CoV-2 linked lockdown, social restrictions impaired the right application of these recommendations, increasing risk of PICS-F, particularly Post-Traumatic Stress Disorder (PTSD).

**Objectives:**

The main objective was to compare the PTSD prevalence at 6 months in a group of relatives including during OLAF implementation with a control group in a cohort of ICU-patient relatives.

**Methods:**

Considering this, the psychiatric team and the ICU team of the Toulouse University, France, proposed the creation of a temporary device called OLAF (*Opération de Liaison et d’Aide aux Familles* in French), aiming to bring a psychological phone support to ICU-patient relatives. Besides this operational approach we designed a research approach that aimed to investigate the impact of OLAF device on PICS-F.

**Results:**

We noted that 12 participants (11.5 %) presented a PTSD at 6 months without statistically significant differences between the groups (p=0.8). Considering that OLAF group presented higher PDI (Peritraumatric Distress Inventory) score at screening we also considered a mediation model suggesting that OLAF could have played a role to diminish the PCL-5 score as a covariable. In the multinomial logistic regression analysis, we found that the only factor associated with the PTSD diagnosis was the level of Anxiety and Depression Signs measured with HADS (Hospital Anxiety and Depression Scale) at screening (OR= 1.2, p<0.001).

**Conclusions:**

We found no difference in PTSD prevalence according to OLAF intervention. Nevertheless, our result suggested that the intervention could have play a role in reducing PTSD by acting on anxiety and peri traumatic distress in a mediated model. We found that anxiety score could serve as a risk marker to predict PTSD and should probably be precociously screened and treated in this population.

**Disclosure of Interest:**

None Declared

